# Exploring the Possibility of a Recovery of Physics Process Properties from a Neural Network Model

**DOI:** 10.3390/e22090994

**Published:** 2020-09-07

**Authors:** Marko Jercic, Nikola Poljak

**Affiliations:** Department of Physics, Faculty of Science, University of Zagreb, 10 000 Zagreb, Croatia; npoljak@phy.hr

**Keywords:** quantum chromodynamics, network model, data analysis, interpretability

## Abstract

The application of machine learning methods to particle physics often does not provide enough understanding of the underlying physics. An interpretable model which provides a way to improve our knowledge of the mechanism governing a physical system directly from the data can be very useful. In this paper, we introduce a simple artificial physical generator based on the Quantum chromodynamical (QCD) fragmentation process. The data simulated from the generator are then passed to a neural network model which we base only on the partial knowledge of the generator. We aimed to see if the interpretation of the generated data can provide the probability distributions of basic processes of such a physical system. This way, some of the information we omitted from the network model on purpose is recovered. We believe this approach can be beneficial in the analysis of real QCD processes.

## 1. Introduction

Modern particle physics has the potential to answer many open fundamental questions, such as the unification of forces, the nature of dark matter or the neutrino masses. To answer these, we turn to data collected by particle accelerators, such as the Large Hadron Collider (LHC) at CERN. These data are collected by detectors which register signals coming from a collision of particles such as protons or lead nuclei. They are almost exclusively complex and of high dimensionality, so untangling them requires a certain level of understanding of the underlying processes that produce them.

The traditional analysis techniques employed in the high energy physics community use sequences of decisions to extract relevant information. The determination of the statistical significance of the extracted quantities then determine if the data yield a new result or not. This approach is usually limited to a single variable, such as the invariant mass of the system. When more than one variable is considered, a multivariate approach is used, which is already a form of a machine learning technique. Lately, a larger number of these techniques are being implemented in high energy physics data analyses, typically including boosted decision trees, genetic algorithms, random forests or artificial neural networks.

This approach to analysis should be natural, since the data resulting from a particle interaction are fundamentally probabilistic due to the quantum mechanical nature of particle collisions. In this sense, the classical approach to data analysis poses a problem because the statistical model describing them can not be known explicitly in terms of an equation that can be analytically evaluated. To make matters worse, even though we have a good model describing the particle interactions (namely quantum chromodynamics), it is inherently non-perturbative and we cannot calculate what it predicts in a certain collision. Therefore, to interpret the collected data we turn to large samples of simulated data generated by stochastic simulation tools such as PYTHIA [1] which try to describe the relevant physics within a nucleus–nucleus collision. However, they have their drawbacks in not being exact, but instead relying heavily on Monte Carlo methods. Even though the knowledge incorporated in the simulators is regularly reinforced with new observations from data, one can never expect the complete physical truth from them.

Considering the fact that we cannot rely entirely on simulated data, we wanted to develop an interpretable model that will provide a way to improve our knowledge of the mechanisms governing particle collisions. We introduce a simple artificial jet generator based only on generalized conservation laws. The simulated data are then passed to a neural network model based only on the partial knowledge of the generator. We try to interpret the generated data and obtain the probability distributions of basic processes of such a physical system, thus recovering some of the information we omitted from the network model. To do this, we make use of the Neyman–Pearson lemma [2], which is an approach that has been proposed by several authors lately [3,4]. Even though we know the model we introduce is a very crude approximation of any real Quantum chromodynamical (QCD) process, with further developments this method could be extended to real data from the LHC, with the hope of gaining new insight on real QCD processes.

This paper is organized as follows: in the Results section we describe how our data are generated and propose the use of the Neyman–Pearson lemma to obtain the underlying probabilities of the data distributions. To do this, we use a neural network classifier and a “guess” dataset. We quantify the differences in the obtained and the original probabilities and present them along with the obtained distributions. In the Discussion section we give a conclusion which follows from these results and present the implications for future research. We conclude the paper with the Materials and Methods section, where we detail the methodology used, should someone want to recreate the results on their own.

## 2. Results

### 2.1. The Jet Generator

To begin with, we create a sample of data based on a simple physical process which will mimic some of the characteristics of the data obtained from particle collider experiments. We start with a particle at rest which decays into two particles. The energies and the momenta of these particles are determined by a selected probability distribution, in this case the distribution of gluon momenta radiated by a quark [5]. The angular distribution of the decay products is selected to be uniform in space.

**Figure 1:**
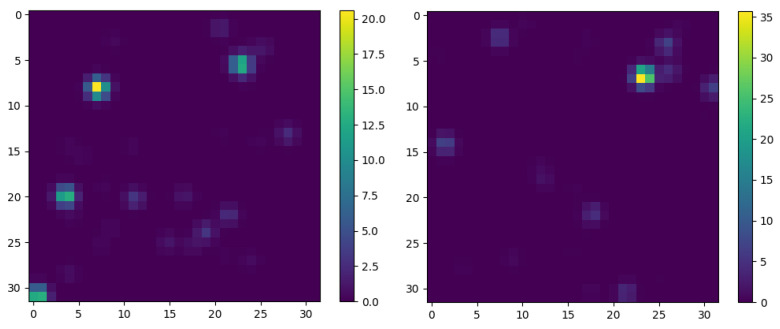
Two examples of jet images generated by the procedure outlined in the text. The *x* and *y* axes of the graphs correspond to the azimuthal angle ϕ and the polar angle θ with respect to the origin. The full solid angle is mapped on these graphs, with 32 bins used for each angle. The color values in these graphs correspond to the energies of the final particles, with the energy of the original particle set to 100. The left panel shows an image of a jet generated with a probability distribution of gluon momenta radiated by a quark. The right panel shows an image of jet generated with a different probability distribution.

After the first decay, the procedure repeats iteratively as described in the Materials and Methods section. The decay procedure stops when either of two conditions is met; if the decay particle mass falls below a preset threshold, or a certain number of decays has been reached. For simplicity, all the decays are considered to happen in the same point in space. The list of final decay particles now forms a *n*-tuple that contains the energies, the momenta and the directions of the *n* particles. We call this entity a jet. To visualize it, we create a histogram whose axes represent the direction of a particle in space. The histogram axes represent the azimuthal angle ϕ and the polar angle θ of a particle. The color of a pixel in the histogram corresponds to either the energy or the momentum of the particle traveling in that direction in space. An example of the jet generator tree with modified parameters is given in Appendix A. Two examples of jet images are given in Figure 1.

The model we chose is simple since it assumes the existence of only one type of particle, which automatically forbids any pair-production channels. This assumption, along with the assumptions of angular uniformity, and the fact that the decay distributions are independent on the invariant mass are not purely QCD like, but we chose to implement them to reduce our computational load.

### 2.2. The Neyman–Pearson Lemma

Let us now forget the decay probability distributions implemented in the data we created. We would like to retrieve them by guessing some of their general characteristics. To do this, we use a neural network to differentiate jet images from the created data and jet images from a “guess” distribution. The idea is the following: first, a number of jets following a known decay probability distribution $p_{\mathrm{real}}$ is created. In our case, this distribution is either the particle energy or the particle momentum distribution, but the arguments we present are valid for any probability distribution. Next, we create another set with the same number of jets in the same manner, this time following a different probability distribution we call $p_{\mathrm{guess}}$.

Assume you are performing a hypothesis test between $H_{0}:p=p_{\mathrm{real}}\left(z\right)$ and $H_{1}:p=p_{\mathrm{guess}}\left(z\right)$ using a likelihood-ratio test. The Neyman–Pearson lemma states that the likelihood ratio, Λ, given by:

(1) $$\Lambda \left(p_{\mathrm{real}}|p_{\mathrm{guess}}\right)\equiv \frac{L(z|p_{\mathrm{real}}\left(z\right))}{L(z|p_{\mathrm{guess}}\left(z\right))}=\frac{p_{\mathrm{real}}\left(z_{1},z_{2},\ldots ,z_{n}\right)}{p_{\mathrm{guess}}\left(z_{1},z_{2},\ldots ,z_{n}\right)}$$

is the most powerful test at a given significance level [2]. Here, $p_{\mathrm{real}}\left(z_{i}\right)$ and $p_{\mathrm{guess}}\left(z_{i}\right)$ are the probabilities associated with the *i*-th decay in a jet having *n* decays in total and following either the $p_{\mathrm{real}}\left(z\right)$ or $p_{\mathrm{guess}}\left(z\right)$ probability distributions.

This means that for a fixed *z*, if we find the most powerful test of distinguishing between jets created following the $p_{\mathrm{real}}$ and $p_{\mathrm{guess}}$ distributions, but we only know $p_{\mathrm{guess}}$, we can recover $p_{\mathrm{real}}\left(z\right)$. This can be done when several assumptions are satisfied. First of all, we consider that all the decays in a decay chain that produces a certain jet are independent. Hence, a jet can be described by a product of factors corresponding to the probability distribution as

(2) $$p\left(z_{1},z_{2},\ldots ,z_{n}\right)=p\left(z_{1}\right)p\left(z_{2}\right)\ldots p\left(z_{n}\right)=p\left(z_{1}\right)p\left(z_{2},\ldots ,z_{n}\right)\;,$$

where $p(z_{i})$ is the probability associated with a single decay in a jet having *n* decays in total. Recall that in this notation $z_{i}$ is a set containing $z_{Ei},z_{pi},\phi_{i}$ and $\theta_{i}$ and the probability $p(z_{i})$ can be written as $p\left(z_{E,i}\right)p\left(z_{p,i}\right)p\left(\phi_{i}\right)p\left(\theta_{i}\right)$. Now let us select the same number of jets generated from $p_{\mathrm{real}}\left(z\right)$ and $p_{\mathrm{guess}}\left(z\right)$ and have a neural network distinguish between them. The neural network is set up as a classifier which gives the probability that the distribution generating an image is $p_{\mathrm{real}}\left(z\right)$, i.e., it gives the value $C_{\mathrm{nn}}\equiv p\left(p_{\mathrm{real}}|z\right)$ [6]. According to the Bayes’ theorem, this value is equal to:

(3) $$\begin{array}{ccc} p(p_{\mathrm{real}}|z) & = & \frac{p\left(z|p_{\mathrm{real}}\right)p\left(p_{\mathrm{real}}\right)}{p\left(z|p_{\mathrm{real}}\right)p\left(p_{\mathrm{real}}\right)+p\left(z|p_{\mathrm{guess}}\right)p\left(p_{\mathrm{guess}}\right)}\\ & = & \frac{p(z|p_{\mathrm{real}})}{p\left(z|p_{\mathrm{real}}\right)+p\left(z|p_{\mathrm{guess}}\right)}=\frac{\Lambda (p_{\mathrm{real}}|p_{\mathrm{guess}})}{\Lambda (p_{\mathrm{real}}|p_{\mathrm{guess}})+1}\;, \end{array}$$

where we take into account the fact that $p\left(p_{\mathrm{real}}\right)=p\left(p_{\mathrm{guess}}\right)$ since we take the same number of jet images from both distributions. By inverting (3) and using (1) and (2), we obtain:

(4) $$\Lambda \left(p_{\mathrm{real}}|p_{\mathrm{guess}}\right)=\frac{C_{\mathrm{nn}}}{1-C_{\mathrm{nn}}}=\frac{p_{\mathrm{real}}\left(z_{1},z_{2},\ldots ,z_{n}\right)}{p_{\mathrm{guess}}\left(z_{1},z_{2},\ldots ,z_{n}\right)}=\frac{p_{\mathrm{real}}\left(z_{1}\right)p_{\mathrm{real}}\left(z_{2},\ldots ,z_{n}\right)}{p_{\mathrm{guess}}\left(z_{1}\right)p_{\mathrm{guess}}\left(z_{2},\ldots ,z_{n}\right)}\;.$$

Now let us look at only $p_{\mathrm{real}}\left(z_{1}\right)$, i.e., the real probability distribution, but for a fixed $z_{1}$. An inversion of (4) gives:

(5) $$p_{\mathrm{real}}\left(z_{1}\right)=\frac{C_{\mathrm{nn}}}{1-C_{\mathrm{nn}}}\cdot p_{\mathrm{guess}}\left(z_{1}\right)\cdot \frac{p_{\mathrm{guess}}\left(z_{2},\ldots ,z_{n}\right)}{p_{\mathrm{real}}\left(z_{2},\ldots ,z_{n}\right)}\;.$$

In our case, this can be applied to $p_{E}\left(z_{E,1}\right)$ and $p_{p}\left(z_{p,1}\right)$ distributions:

(6) $$\begin{array}{ccc} p_{\mathrm{real},E}\left(z_{E,1}\right) & = & \frac{C_{\mathrm{nn}}}{1-C_{\mathrm{nn}}}\cdot p_{\mathrm{guess},E}\left(z_{E,1}\right)\cdot \frac{p_{\mathrm{guess},p}\left(z_{p,1}\right)p_{\mathrm{guess}}\left(z_{2},\ldots ,z_{n}\right)}{p_{\mathrm{real},p}\left(z_{p,1}\right)p_{\mathrm{real}}\left(z_{2},\ldots ,z_{n}\right)}\;\mathrm{and}\\ p_{\mathrm{real},p}\left(z_{p,1}\right) & = & \frac{C_{\mathrm{nn}}}{1-C_{\mathrm{nn}}}\cdot p_{\mathrm{guess},p}\left(z_{p,1}\right)\cdot \frac{p_{\mathrm{guess},E}\left(z_{E,1}\right)p_{\mathrm{guess}}\left(z_{2},\ldots ,z_{n}\right)}{p_{\mathrm{real},p}\left(z_{E,1}\right)p_{\mathrm{real}}\left(z_{2},\ldots ,z_{n}\right)}\;. \end{array}$$

This final expression offers a possibility of recovering $p_{\mathrm{real},E}$ and $p_{\mathrm{real},p}$ by only knowing $p_{\mathrm{guess},E}$ and $p_{\mathrm{guess},p}$ in the case where the neural network acts as an ideal classifier. It is assumed that all of the angles occur with equal probabilities so they are omitted from the equation. To recover the real probability distribution, we used a feed forward convolutional neural network (CNN) [7]. The inputs used for the network are jet images, the examples of which can be seen on Figure 1, while the output it gives is $C_{\mathrm{nn}}$, the parameter most relevant to our calculations. The architecture and the details of the used network are given in the Materials and Methods section.

### 2.3. Recovering the Original Probability Distribution

In what follows, the indices *E* and *p* are omitted to improve clarity, but the general conclusions work for either the energy distribution $p_{\mathrm{guess},E}$ or the momentum distribution $p_{\mathrm{guess},p}$. To provide a reasonable $p_{\mathrm{guess}}$ distribution, we have to know some of the background of the physical process that governs $p_{\mathrm{real}}$. For example, from our physics background we know that this distribution should fall with increasing *z*. An example of such a distribution is

(7) $$p_{\mathrm{guess}}\left(z\right)=Ne^{-Cz}\;,$$

whose integral is normalized to 1. This distribution is allowed to be only “good enough” when using the outlined procedure, since we can iteratively repeat it and set

(8) $$p_{\mathrm{guess}}^{i+1}\left(z\right)=p_{\mathrm{real},\mathrm{calculated}}^{i}\left(z\right)\;,$$

with *i* being the iteration index and $p_{\mathrm{real},\mathrm{calculated}}^{i}\left(z\right)$ being the approximation of the “real” distribution as determined in the current step. The reason why the guess distribution converges to the real distribution when applying this procedure iteratively can be seen if one looks at the cross entropy loss of the neural network. This quantity, also known as the log loss, measures the performance of a classification model where the prediction input is a probability value between 0 and 1 [8]. In the case of binary classification, which we perform here, and using the notation already given in the text, it is given by:

(9) $$L=-\frac{1}{2}\sum_{i=1}^{n}\left[y\left(z_{i}\right)\log C_{\mathrm{nn}}+\left(1-y\left(z_{i}\right)\right)\log\left(1-C_{\mathrm{nn}}\right)\right]\;.$$

where $y(z_{i})$ is the set of true data labels, being either 1 or 0, depending on which distribution was used to create a particular jet. In general, the performance of any model is always worse compared to the ideal model, so that the cross entropy loss of our classifier *L* has to be larger than the loss of an ideal classifier $L_{\mathrm{ideal}}$. Using (3), this can be written as:

(10) $$\begin{array}{ccc} L & > & -\frac{1}{2}\sum_{i=1}^{n}\left[y\left(z_{i}\right)\log C_{\mathrm{nn}}^{\mathrm{ideal}}+\left(1-y\left(z_{i}\right)\right)\log\left(1-C_{\mathrm{nn}}^{\mathrm{ideal}}\right)\right]\\ & > & -\frac{1}{2}\sum_{i=1}^{n}\left[y\left(z_{i}\right)\log\left(\frac{\Lambda (p_{\mathrm{real}}|p_{\mathrm{guess}})}{1+\Lambda (p_{\mathrm{real}}|p_{\mathrm{guess}})}\right)+\left(1-y\left(z_{i}\right)\right)\log\left(\frac{1}{1+\Lambda (p_{\mathrm{real}}|p_{\mathrm{guess}})}\right)\right] \end{array}$$

Now let us assume that the classifiers have been fed only the data from the real distribution, i.e., that we set $p_{\mathrm{guess}}^{i}=p_{\mathrm{real}}$ on purpose. Then the data labels $y(z_{i})$ are all equal to 1, so that

(11) $$-\frac{1}{2}\sum_{i=1}^{n}\log C_{\mathrm{nn}}>-\frac{1}{2}\sum_{i=1}^{n}\left[\log\left(\frac{\Lambda (p_{\mathrm{real}}|p_{\mathrm{guess}})}{1+\Lambda (p_{\mathrm{real}}|p_{\mathrm{guess}})}\right)\right]\;.$$

Although the index *i* has been left out to improve readability, both expressions under the sum still depend on the selected *z*-bin. A short rearrangement of this condition gives:

(12) $$\prod_{i=1}^{n}C_{\mathrm{nn}}<\prod_{i=1}^{n}\frac{\Lambda (p_{\mathrm{real}}|p_{\mathrm{guess}})}{1+\Lambda (p_{\mathrm{real}}|p_{\mathrm{guess}})}\;.$$

Now we use the fact that $C_{\mathrm{nn}}>0.5$, which we know to be true averaged over *z*, if the network has any discriminating power. Using (3) again, the last inequality can be rearranged into:

(13) $$\prod_{i=1}^{n}\frac{C_{\mathrm{nn}}}{1-C_{\mathrm{nn}}}<\prod_{i=1}^{n}\Lambda \left(p_{\mathrm{real}}|p_{\mathrm{guess}}\right)\;,$$

Note that the left side of this inequality is a product larger than one, even though some of the factors after the product sign can be smaller than one. Recalling the definition of $\Lambda (p_{\mathrm{real}}|p_{\mathrm{guess}})$, after multiplying with $p_{\mathrm{guess}}$ we can write:

(14) $$\prod_{i=1}^{n}p_{\mathrm{guess}}\left(z_{i}\right)<\prod_{i=1}^{n}\frac{C_{\mathrm{nn}}}{1-C_{\mathrm{nn}}}p_{\mathrm{guess}}\left(z_{i}\right)<\prod_{i=1}^{n}p_{\mathrm{real}}\left(z_{i}\right)\;.$$

The first term on the left is the guess distribution in one of the iterations, the second term is the next iteration of the guess distribution since we are using (6) and (8) and the last term is the real distribution. Thus, we can conclude that in this case, the iterations successively converge to the real distribution. The same argument can be used when the network is fed only the data from the guess distribution. Since the real data are a mix of the two we conclude that in general, the successive iterations of the guess distribution converge on average to the real distribution. If we could perform an infinite number of iterations, we would reach the real distribution from the guess distribution, but since we have limited time and resources, the two will always be at least slightly different.

### 2.4. Calculation Results and Errors

Our calculation was performed with the initial guess probability distributions given by $p_{\mathrm{guess},E}=p_{E}^{0}\left(z_{E}\right)=N_{E}e^{-Cz_{E}}$, with $z_{E}$ in the interval [0.01,0.5] and $p_{\mathrm{guess},p}=p_{p}^{0}\left(z_{p}\right)=N_{p}e^{-Cz_{p}}$ with $z_{p}$ in the interval [0.01,1]. Three different values of the constant *C* were used, 0.1, 10 and 100, thus creating a nearly flat distribution, a distribution slowly decreasing with increasing *z* and a much more rapidly decreasing distribution, respectively.

Once the iterative procedure starts, we need to decide at which point to stop further iterations. We define the error margin of the *i*-th iteration for a single variable (either energy or momentum) of a guess distribution as the root mean square relative error (RMSRE), which is a typical cross-validation tool [9]:

(15) $$\mathrm{RMSRE}=\sqrt{\frac{1}{10}\sum_{j=1}^{10}{\left(1-\frac{p_{\mathrm{guess}}^{i}\left(z_{j}\right)}{p_{\mathrm{real}}\left(z_{j}\right)}\right)}^{2}}\;.$$

The index *j* comes from the fact that we had to choose a number of *z* bins, which we set to 10, in order to perform the calculations. The total error margin for an iteration of a guess distribution is defined as the arithmetic mean of the margins for energy and momentum. We stop the iterative procedure once the error margin remains below 10% during 20 successive iterations [10].

**Figure 2:**
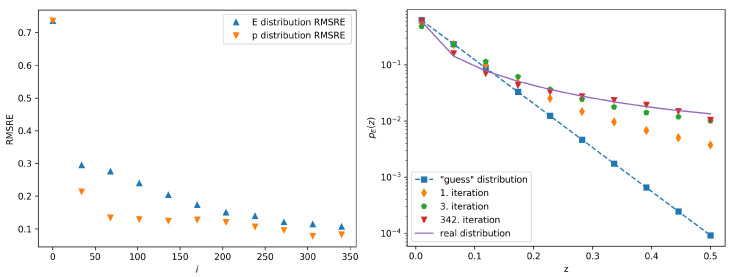
The left panel shows the calculated error margin vs. the iteration number in the case of the guess distribution given by (7) with *C* set to 10. The error calculation is described in the text. The error margins are shown separately for the case when the classifier is trained with jet images populated either with jet energies or jet momenta. The right panel shows Several iterations of the calculated probability distributions $p_{E}^{i}\left(z\right)$ (symbols) compared to $p_{\mathrm{real},E}\left(z\right)$ (full line). The 342nd iteration is the final iteration of this procedure, since the stopping condition has been satisfied.

A graph showing the dependence of the error margin on the iteration index for the case of distribution (7) with *C* set to 10 is given in Figure 2. The graph shows the margins for the calculated $p_{E}^{i}\left(z\right)$ and $p_{p}^{i}\left(z\right)$ distributions. In this case the average error margin is lower than 10% for 20 successive iterations after the 342nd iteration. On the same figure we also show the calculated probability distributions $p_{E}^{i}\left(z\right)$ and compare them to $p_{\mathrm{real},E}\left(z\right)$. The comparison of the probability distributions $p_{p}^{i}\left(z\right)$ to $p_{\mathrm{real},p}\left(z\right)$ for different parameters *C* is given in the Appendix B.

**Figure 3:**
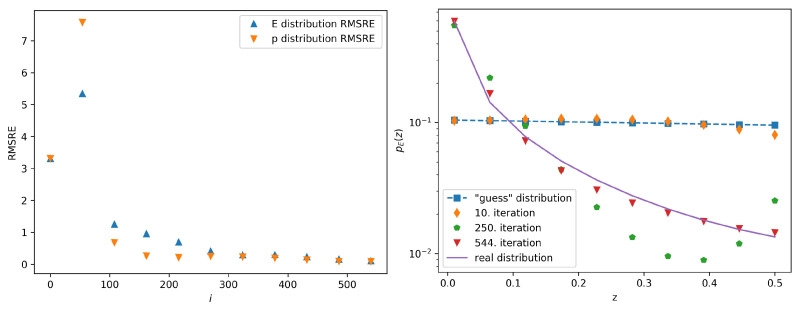
The left panel shows the calculated error margin vs. the iteration number in the case of the guess distribution given by (7) with *C* set to 0.1. The right panel shows several iterations of the calculated probability distributions $p_{E}^{i}\left(z\right)$ (symbols) compared to $p_{\mathrm{real},E}\left(z\right)$ (full line). The 544th iteration is the final iteration of this procedure, since the stopping condition has been satisfied.

**Figure 4:**
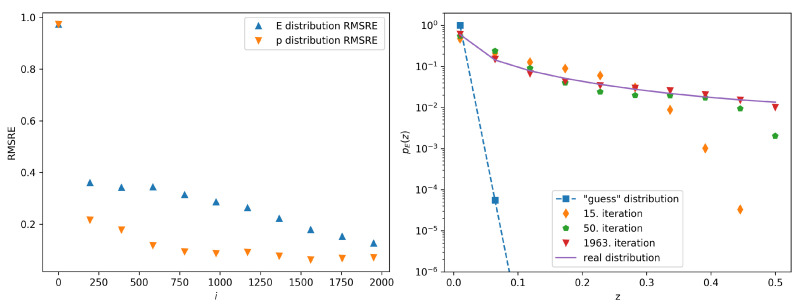
The left panel shows the calculated error margin vs. the iteration number in the case of the guess distribution given by (7) with *C* set to 100. The right panel shows several iterations of the calculated probability distributions $p_{E}^{i}\left(z\right)$ (symbols) compared to $p_{\mathrm{real},E}\left(z\right)$ (full line). The 1963rd iteration is the final iteration of this procedure, since the stopping condition has been satisfied.

One can see the decrease of the error margin with growing iteration index and the convergence of the guess distribution to the real distribution. The graphs showing the dependence of the error margins on the iteration index and the calculated probability distributions $p_{E}^{i}\left(z\right)$ compared to $p_{\mathrm{real},E}\left(z\right)$ when *C* equals 0.1 and 100, respectively, are given in Figure 3 and Figure 4. In these cases, the stopping condition has been reached after 544 and 1963 iterations, respectively. When comparing the results for different initial guess distributions, we note that only the total number of the iterations needed to achieve sufficient convergence is affected by the initial conditions. It is interesting to note that during some of the iterations the distribution is no longer monotonically decreasing, as can be seen on Figure 4 for the 250th iteration. This happens due to the finite sample and numerical rounding, but the final distribution nevertheless converges into the real distribution.

## 3. Discussion

In this paper we present a study performed on a toy model representing a crude version of a QCD fragmentation process. It is possible to retrieve some of the unknown properties of this process by using a correct interpretation of a neural network model combined with incomplete knowledge of the system. We presented an iterative method which recovers unknown probability distributions that govern the presented physical system. We have mathematically shown that one can expect the convergence from our incomplete knowledge to the real underlying distributions by using the developed method. This claim was confirmed by our results.

The method we chose requires an initial guess of the probability distributions from which the original distributions are to be recovered. The choice of the guessed probability distributions affects only the number of iterations needed to achieve the convergence to the real distributions. The final error margin between the obtained distributions and the real distributions should depends only on the discriminating power of the used classifier, i.e., the convolutional neural network. In our study, we used a stopping condition which relies on the RMSRE between the real and the calculated distribution. However, this relies on the fact that we constructed and knew the real distribution, which is not true in a realistic setting. In that case, the stopping condition could be based solely on the the loss function of the classifier, evaluated on some test dataset. For example, one could impose the condition that the values of the loss function are in some small interval around the minimal possible loss value $L_{\min}$. In that case the expected values of the classifier output will lie in some small interval around $C_{\mathrm{nn}}=0.5$ and any further calculation will not significantly improve the probability distributions obtained in the previous iteration.

Since this method does not imply what kind of classifier should be used, any machine learning technique used for binary classification can be employed. In this research we developed a classifier based on convolutional neural networks, which have proven to be very successful in the image classification tasks. We believe that the presented method can be generalized for use in more realistic physical systems which include multiple decay mechanisms. For example, we could also introduce the dependence of the probability distributions on the current invariant mass, or the case when the polar angle is not uniformly distributed. The correlation between the energy and the angle, which is present in reality, could also be studied by looking at joint distributions, instead of looking each of the distributions independently. Surely, these modifications would bring us closer to a real QCD process. However, in this paper we only wanted to perform the first step in showing that some characteristics of the process can be retrieved with the help of a neural network. If one were to develop the method further, increasing the similarity to QCD, we believe it could be applied to real data collected by some high energy experiment. A possible way to go about this would be to perform a similar analysis on an existing model, such as data from PYTHIA. This is computationally demanding and we leave it for future research.

## 4. Materials and Methods

In this section, we present in detail the methodology used to obtain the presented results. First, we describe the jet generator used to crate the jet images. Next, we present the detailed architecture of the neural network used as the classifier and finally, we detail the algorithm used to recover of the underlying probability distributions.

The computational code used to develop the particle generator, the neural network model and the calculation of the probability distributions is written in the the Python programming language using the Keras module with the TensorFlow backend [11]. Both the classifier training and jet generating were performed using a standardized PC setup equipped with an NVIDIA Quadro p6000 graphics processing unit.

### 4.1. The Jet Generator

1. We start with a particle at rest with a given rest mass, here taken to be $m_{0}=\;$100 (the units are inconsequential in the calculation).
2. The particle decays into two new particles. The energies and the momenta of these particles are determined by a probability distribution. To generate the real data we use a distribution already known in particle physics, given by:
  (16) $$p\left(z\right)=N\frac{1+{\left(1-z\right)}^{2}}{z}\;.$$
  The energy of the decay particle *E* equals $zE_{0}$, with $E_{0}=m_{0}$ being the energy of the decaying particle. Note that the probability diverges as *z* approaches zero, so the distribution is limited by a lower boundary on *z* both due to physical and computational reasons. N is a constant that ensures that the integral of the probability distribution equals 1 and depends on the lower boundary set on *z*. In our simulation, we set the minimum *z* to 10${}^{-2}$, making N equal to ≈0.13.
  The momentum of the decay particle is limited with the total energy of the particle. We determine the momentum by sampling the same probability distribution as for the energy, but now we set the momentum *p* equal to zE, with *E* being the energy of the decay particle. To differentiate between these *z* distributions, we write $z_{E}$ and $z_{p}$ when deemed necessary.
  The spatial distribution of the decay products is uniform in space. This means that, observed from the rest frame of the decaying particle, the probability that either one of the decay products flies off in a certain infinitesimal solid angle is uniform. Physically speaking, the angles θ and ϕ are sampled from uniform distributions on intervals [0,π] and [0,2π], respectively.
  The energy, the momentum and the direction of the second particle are determined by the laws of conservation of energy and momentum. In other words, $z_{1}+z_{2}=1$ when looking at energy, and $p_{1}+p_{2}=0$, since the original momentum in the center of mass system is zero. These facts also save computational time due to symmetry, since we can sample for the energy of the first particle in the interval $\left[0.01,0.5\right]$, instead of placing the upper limit for *z* to 1.
3. After the first decay, the procedure repeats iteratively, i.e., we repeat step 2 for both decay products from the previous step. The only difference compared to the previous step is that we now perform the calculations for each particle in its center of mass frame and then transform the obtained quantities back to the laboratory frame, which coincides with the center of mass frame of the original particle.
  Once the total number of particles exceeds a pre-determined threshold (in our case set to 32), we disregard the lowest energy particles. We do this both to reduce the computational time and because we determined that these particles do not influence our end result in a significant manner.
  The decay procedure stops when either of two conditions is met; if the decay particle mass falls below 0.1, or a certain number of decays has been reached. In the simulations, we limited the number of decays in a single branch to 50. For simplicity, all the decays are considered to happen in the same point in space.
4. The list of final decay particles now forms a list that contains the energies, the momenta and the directions of the *n* particles. We call this entity a jet. The jet has a maximum of 32 particles in its final state stemming from a maximum of 1 + 2 + 4 + 8 + 16 + 45·32 = 1471 decays. Hence, the full description of a jet is given by a maximum of 1471 $z_{E}$ parameters, 1471 $z_{p}$ parameters and 1471 pairs of angles (θ, ϕ).
  To create the final representation of the jet which will be fed to a classifier, we create a histogram whose axes represent the direction of a particle in space. The histogram has 32 × 32 pixels with axes representing the polar angle θ and the azimuthal angle ϕ of a particle. The color of a pixel in the histogram corresponds to either the energy or the momentum of the particle traveling in that direction in space. We distribute the deposited energy and momentum as Gaussian distributions in the histograms, with the Gaussian of σ equal to 1 pixel centralized at the pixel corresponding to a direction of a certain particle. This mimics the physics situation in real life, where the readout from a detector always consists of a signal and a background noise. In fact, even when simulating data in a deterministic way, this effect is taken into account [12]. Lastly, the energy and momentum histograms are stacked to create an image with dimensions 32 × 32 × 2. An example of the jet generator tree with modified parameters is given in the appendix. Two examples of jet images are given on Figure 1 in the main body of the text.

### 4.2. The Classifier

**Figure 5:**
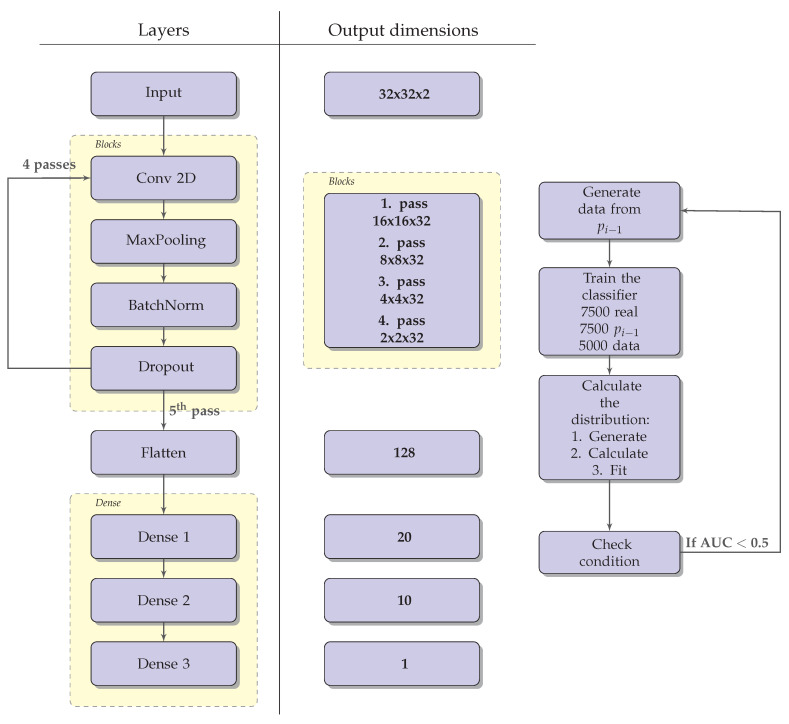
The left panel shows the architecture of the convolutional neural network as described in the text. The output dimensions of each layer are given on the right side of the panel. The Blocks layer goes through 4 passes. The right panel shows the algorithm used to recover the underlying probability distributions. AUC stands for Area Under the Curve and provides an aggregate measure of the network performance.

The classifier used to recover the real probability distribution is a feed forward convolutional neural network (CNN). The architecture of the used CNN is schematically shown on Figure 5. It consists of a block of layers, repeated four times, followed by 3 dense layers consisting of 20, 10 and 1 unit, respectively. A rectified linear activation function is used in each layer, except for the last one, where a sigmoid function is used. The layer block consists of a 2-dimensional convolutional layer (with 32 filters and a (3,3) kernel), a MaxPooling layer, a batch normalization layer and a dropout layer. The training of the classifier is performed by minimizing the binary cross entropy loss [8]. The AdaM optimizer is used to optimize the weights of the CNN [13]. When training through the iterations, in each iteration we use the same number of jets obtained with $p_{\mathrm{real}}\left(z\right)$ and jets obtained by using the distribution calculated from the previous iteration. To train the CNN we used 75% of data, while the remaining 25% were used to validate the trained model.

### 4.3. The Algorithm Used to Recover the Underlying Probability Distributions

After the real jets dataset was generated, we wanted to recover its underlying probability distributions, which we treated as unknown. The schematic view of the algorithm we use for this is shown on Figure 5. The algorithm is repeated iteratively. To begin with, we set some initial guesses of the underlying distributions, denoted by $p_{E}^{0}\left(z_{E}\right)$ and $p_{p}^{0}\left(z_{p}\right)$. Each iteration, indexed by *i*, consists of 3 steps: first, the data are generated using the probability distributions $p_{E}^{i}\left(z_{E}\right)$ and $p_{p}^{i}\left(z_{p}\right)$. Next, the classifier is trained on the generated data and then the probability distributions $p_{E}^{i+1}\left(z_{E}\right)$ and $p_{p}^{i+1}\left(z_{p}\right)$ are calculated using the trained classifier. After each iteration the weights of the classifier are saved and used as the initial weights for the training procedure in the next iteration. The iterative procedure is stopped once the error margin (15) remains below 10% during 20 successive iterations. Further subsections present the details of the outlined algorithm.

#### 4.3.1. Generating the Data From the Obtained Distributions

To generate the data used for the next iteration we sample 10,000 vectors $z\equiv (z_{1},z_{2},\ldots ,z_{N})$, where $N_{\max}=1471$ and $z_{n}\equiv \left(z_{E}^{n},z_{p}^{n},\theta^{n},\phi^{n}\right)$. The parameters $z_{E}$ and $z_{p}$ are sampled from the $p_{E}^{i}\left(z_{E}\right)$ and $p_{p}^{i}\left(z_{p}\right)$ probability distributions obtained from the previous iteration. These vectors are fed into the jet generator to obtain 10,000 jet images.

#### 4.3.2. Training the CNN Classifier

The generated data are next used to train the classifier. The 10,000 samples of jets generated by the distributions $p_{E}^{i}\left(z_{E}\right)$ and $p_{p}^{i}\left(z_{p}\right)$ are paired with 10,000 randomly chosen samples from the dataset containing jets generated using the real distributions. If the $p_{E}^{i}\left(z_{E}\right)$ and $p_{p}^{i}\left(z_{p}\right)$ distributions and the real distributions are very different, the output of the classifier $C_{\mathrm{nn}}$ can be expected to be very close to 0 or 1. This can occur during the early iterations of the algorithm and can cause computational difficulties due to nature of the denominator in (6). To avoid these difficulties, the classifier was trained on a smaller dataset during the early iterations, typically containing ≈ 200–2000 jets.

#### 4.3.3. Calculation of the Probability Distributions

In order to calculate $p_{E}^{i+1}\left(z_{E}\right)$ and $p_{p}^{i+1}\left(z_{p}\right)$, we use (6). First, we generate a vector $z\equiv (z_{1},z_{2},\ldots ,z_{N})$, where $z_{n}=\left(z_{E}^{n},z_{p}^{n},\theta^{n},\phi^{n}\right)$, by sampling the $p_{E}^{i}\left(Z_{E}\right)$ and $p_{p}^{i}\left(z_{p}\right)$ probability distributions. From each of these vectors we remove $z_{E}^{1}$ and fix it by hand to a value between 0.01 and 0.5 in 1000 equidistant bins. This way, we create 1000 vectors *z* which differ only in the $z_{E}$ parameter of the first decay. Our jet generator is then used to create the jet images. Each of the images is fed to the classifier, which gives us $C_{\mathrm{nn}}^{j}\left(z_{j}\right)$, with *j* being the index of the image. The second term in (6) can be directly calculated using the $p_{E}^{i}\left(z_{E}^{i}\right)$ distribution. The last two terms form a constant which is equal for all of the used jet images. The calculation of the constant is simple: since we are dealing with probability distributions, we impose the condition that the integral $p_{E}\left(z_{E}\right)$ over $z_{E}$ equals 1, which directly determines the value of the constant. This way, we obtain the value of the probability distribution $p_{E}\left(z_{E}\right)$ for each $z_{E}$ bin. This procedure is repeated 200 times with jet images generated with different decay conditions. The arithmetic average of the calculated distributions is used to finally determine the distribution $p_{E}^{i+1}$. Due to the nature of the algorithm, $p_{E}^{i}\left(z_{E}\right)$ inevitably will not be a smooth function since it is calculated on a point to point basis. Before feeding this distribution to the next iteration, we perform a smoothing by fitting a fourth degree polynomial to the calculated values on a log scale. An analogous procedure is used to determine $p_{p}^{i}\left(z_{p}\right)$. The only difference is that instead of $z_{E}$, in this case we fix the $z_{p}$ parameter of the first decay.

## Figures and Tables

**Figure A1:**
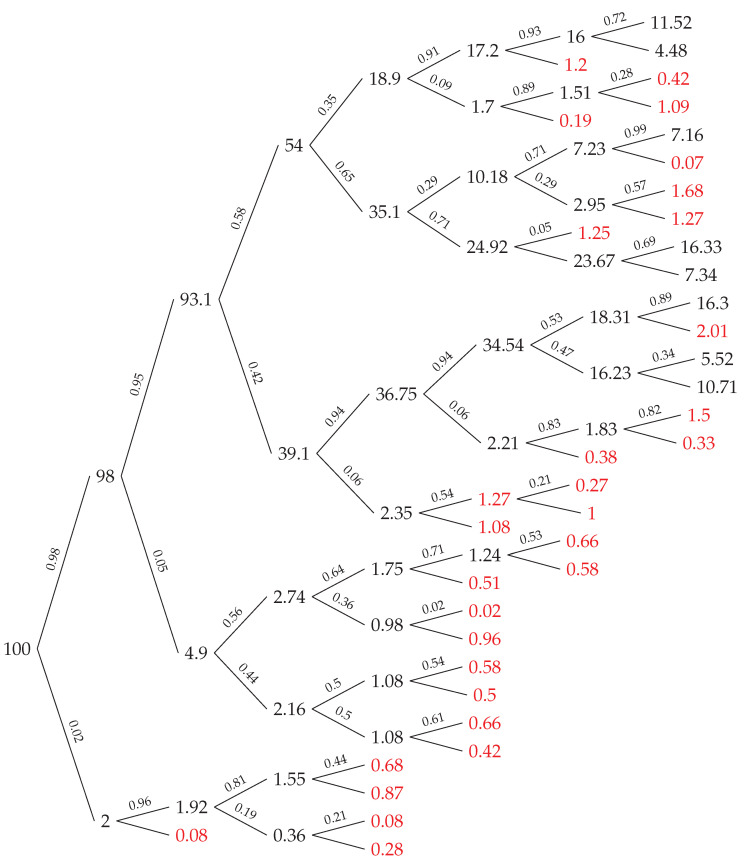
An example of the operation of the jet generator. The number on the specific node represents the total energy for a given particle, while the number on the line connecting two nodes is the energy ratio *z* when decaying. The decay probability distribution p(z) in this image is constant. The maximum number of decays in a single branch has been set to 7, and the maximum number of particles in the jet has been set to 8. A particle stops to decay once its energy is too low (here set to 0.1). The particles coloured red are removed from the jet because their energy is too low.

**Figure A2:**
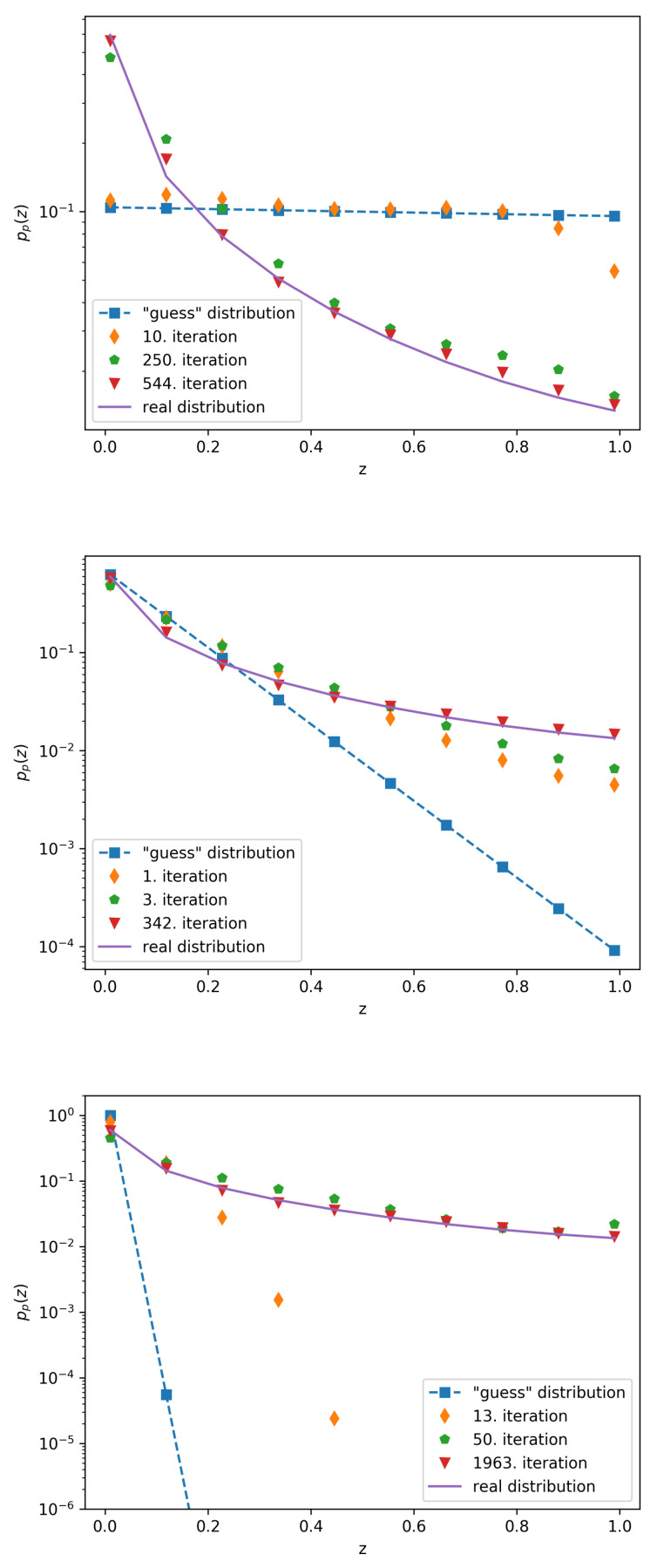
Several iterations of the calculated probability distributions $p_{p}^{i}\left(z\right)$ (symbols) compared to $p_{\mathrm{real}}\left(z\right)$ (full line) in the case of the guess distribution given by (7). Top: C=0.1. Middle: C=10 and Bottom: C=100.

## Abbreviations

The following abbreviations are used in this manuscript:

QCD	Quantum Chromodynamics
LHC	Large Hadron Collider
RMSRE	Root mean square relative error
CNN	Convolutional Neural Network
AUC	Area Under the Curve

## Appendix A. An Example of a Generated Jet

Here we give a pictorial example of a jet generated as outlined in Section 2 (see Figure A1).

## Appendix B. Supplementary Results

Here we give the comparison of momentum probability distributions $p_{p}^{i}\left(z\right)$ when varying the parameter *C* as a complement to the results for $p_{E}^{i}\left(z\right)$ given in the text (see Figure A2).
